# Effects of the Human Amniotic Membrane on the Cartilage Graft: Prognosis and Absorption in White Rabbits

**DOI:** 10.29252/wjps.8.2.219

**Published:** 2019-05

**Authors:** Sadrollah Motamed, Peyman Mohammadi Torbati, Hamid Zaferani Arani, Amir Reza Motabar, Amirhossein Zabolian, Zahra Madadi

**Affiliations:** 1 Department of Plastic Surgery, Shahid Beheshti University of Medical Sciences, Tehran, Iran;; 2 Department of Pathology, Shahid Beheshti University of Medical Sciences, Tehran, Iran;; 3 Young Researchers and Elite Club, Tehran Medical Sciences, Islamic Azad University, Tehran, Iran;; 4 Department of Plastic Surgery, Tehran Medical Sciences, Islamic Azad University, Tehran, Iran;; 5 Non-communicable Diseases Research Center, Endocrinology and Metabolism Population Sciences Institute, Tehran University of Medical Sciences, Tehran, Iran;; 6 Endocrinology and Metabolism Research Center, Endocrinology and Metabolism Clinical sciences Institute, Tehran University of Medical Sciences, Tehran, Iran

**Keywords:** Graft, Cartilage, Amniotic membrane, Rabbit

## Abstract

**BACKGROUND:**

Cartilage grafts are generally accepted for the restoration and reconstruction of nasal contours. The main concern that plastic surgeons may need to address after surgery pertains to the resorption and disfigurement of the grafted cartilage, especially in allogenic and heterogenic grafts.

**METHODS:**

A total of 12 white rabbits were divided into three groups according to the types of graft including autograft, allograft, and heterograft. We used three shapes of grafts, including block, crushed, and diced cartilage in the upper, middle, and lower rows. However, in each rabbit, these grafts were divided into two columns of wrapped and unwrapped grafts, with human amniotic membrane (HAM) grafted on each side of the rabbit’s back.

**RESULTS:**

In total, 60 specimens underwent histopathological examination. No inflammation was observed in about 50% of the block-shaped conchal cartilages with HAM, and in 50%, less than 25 inflammatory cells per unit were seen. The prognosis and absorption of autograft specimens in block-shaped cartilages with HAM were significantly better compared with other shapes of cartilages with HAM and without HAM. The proliferation rate of fibroblasts in autograft and allograft specimens was more than that in heterograft specimens with HAM.

**CONCLUSION:**

Our findings have demonstrated the new role of HAM in clinical applications, indicating that HAM may be used as a low-cost, easily accessible alternative for wrapping in cartilage grafts instead of fascia or surgicel in early future. It is useful for improving the long-term outcomes and decreasing the resorption rate.

## INTRODUCTION

Grafts are available in several types, including autograft, allograft, and heterograft.^1^ The ideal source for all kinds of grafts in rhinoplasty is the autogenous cartilage, particularly the septum of nose that is widely considered by most plastic surgeons^1^^,^^2^ for nasal amplification because of the lowest infection rate, minimal immune system stimulation, biocompatibility, and undermost donor site morbidity associated with its use compared with other types.^3^ Nevertheless, one of the major drawbacks of this type of graft is restricted access to source it, especially in secondary and tertiary rhinoplasty operations. For this reason, many surgeons use alternative sources such as fascia, fat, dermis, and mesh. However, these materials have some disadvantages, including shrinkage, resorption, atrophy, distortion, need for overcorrection, and infection. These are reasons that there is no common agreement with regard to the clinical application of these materials.^4^^-^^9^

Durability and viability of various forms of cartilage grafts and usage of wrapped or bare graft were studied by some authors.^9^ Although some studies have shown the long-term prognosis of the irradiated allograft cartilage and adipose-derived stem cells in nasal reconstruction, no solution with respect to the improved prognosis of the graft has yet been accepted by the plastic surgeon community.^10^Applications of these materials in cases of depletion of autogenous cartilage have not been accepted generally. As a result of this conflicting information and difference in resorption rate, application of stem cells for restore and improve the graft outcome is new concept and backbone of our research.^11^

Recently, the human amniotic membrane (HAM) and human umbilical cord have been found as a source of the mesenchymal stem cell (MSC) that is bioequivalent to bone marrow MSC.^12^ Its vast applications as biological coverage and for promoting healing of corneal ulcers are clearly understood now with significant effects.^13^ Human amniotic stem cells (HASCs) are an interesting example of allogenic cells that are currently used for tissue engineering. HASCs possess high levels of telomerase activity and express the surface markers, SSEA-4, TRA (tumour rejection antigen)-1-60 and TRA-1-81.^14^ In addition, they also show high expression of octamer binding protein 4 (Oct-4) and Nanog.^14^^,^^15^

Using these HASCs, researchers have been successful in generating cells of ectodermal, endodermal, and mesodermal lineage. This differentiation was determined using the formation of embryoid bodies in vitro and teratomas in vivo. Teratomas form when embryonic stem cells are injected into severe combined immunodeficient (SCID) mice and tissue types formed include gut epithelium, cartilage, bone and neural epithelium among others.^16^ However, some questions remain unresolved regarding this technique, such as the way that the transplanted stem cells effect healing and whether autologous or allologous stem cells are the most effective for transplantation.^17^

The combined application of both cell sources (MSCs and HAM) is expected to have wide clinical use such as improvement of cartilage graft viability, and graft architecture. The idea of how to improve the prognosis and reduce the resorption rate or prevention of disfigurement of the grafts, especially allogenic or heterogeneous grafts, has been the main concern of plastic surgeons. The definitive solution has not presented yet. Over the last decade, many reports have supported the isolation of pluripotent or multipotent stem cells from human placenta cord blood or amniotic fluid.^17 ^The aim of this study was to observe and compare the effects of HAM as a source of easy access stem cells on prognosis, cartilage graft viability, and graft architecture, as well as the outcome of different kinds and shapes of cartilage grafts. 

## MATERIALS AND METHODS

For this experimental study, 15 white rabbits weighing between 1500 and 2000 g and aged about 15 months were selected. Fifteen rabbits were divided into three groups randomly. Three rabbits died before the end of the study (1 rabbit in each group) and were thus excluded from all analyses. Group 1 used the autogenous graft, group 2 the allogenous graft, and group 3 the heterogeneous graft. The allogenous grafts were obtained from the rabbits’ ears in group 1, and in the heterogeneous group, lyophilized human cadaveric nasal septum were used as a standard product that processed in Shahid Beheshti University of Medical Sciences (The Academic Center for Education, Culture and Research (ACECR)) which was easily accessible and had routinely clinical apply. For the method of general anesthesia, each rabbit was administered an injection of ketamine hydrochloride (40 mg/kg, Sigma-Aldrich, USA). 

Fresh HAM was obtained with written informed consent from healthy full-term women after uncomplicated cesarean deliveries in Taleghani General Hospital, Tehran, Iran. Women with any history of infectious disease and other high-risk pregnancies were excluded from this study. The placentas and chorions after harvesting were placed in a plate with some types of antibiotics with appropriate doses.^18^ The amnion layer was mechanically peeled off from the chorion under stringent sterile conditions and washed with normal saline (0.9%) for four to six times to remove all clots, mucous, and debris. The thin and transparent layer of HAM was separated from the chorion of the placenta and kept in wet and sterile gauze. Thereafter, it was transferred to the animal laboratory in a sterile container and applied within 6 hours. The Ethics Committee of Shahid Beheshti University of Medical Sciences approved the technical steps of this research and approved this study with ethics code of SBMU.REC.1393.606. 

**Fig. 1:**
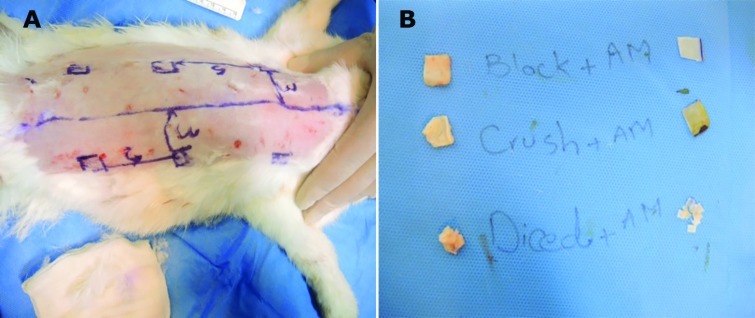
Illustrations of the methodology for preparation and placing of cartilages. Six subcutaneous pockets were created 3 cm from back midline and 5 cm from each other (A). Right column wrapped and left column is bare grafts. Upper row block grafts, middle row crushed grafts that made by hemostate and inferior row diced grafts (B)

The skin on the rabbit’s back and paraspinal area was shaved, cleansed with povidone iodine (Betadine; Purdue Pharma LP, Stamford, Connecticut) solution, and draped in a sterile manner. After the injection of diluted epinephrine (1:100,000) with the subcutaneous area was infiltrated with lidocaine hydrochloride (1%), 1.5-cm skin incisions were made three on the right side and three on the left side of the back paraspinal area, and then small subcutaneous pockets were created away from the midline (Figure 1A). 

The six (10×10)-mm grafts without prechondrium, in three different shapes, that is, block, crushed, and diced, were implanted in pockets. We put block, crushed, and diced grafts in the upper, middle, and lower rows, respectively. Half of the grafts were wrapped with a piece of (25×25)-mm HAM. The wrapped grafts were located on the right side of each rabbit. The bare cartilage grafts of the left side were considered as control groups (Figure 1B). The 6 grafts were implanted into the 6 subcutaneous pockets and were sutured using 5/0 polypropylene sutures (Prolene; Ethicon Inc, Somerville, New Jersey) on the dorsum. 

After the implantation of 72 grafts, skin closure was completed using interrupted silk sutures 3/0 (Vicryl; Ethicon Inc) on the dorsum. Tetracycline ointment was used on each suture line for 2 days and postoperative 50 mg/kg ceftriaxone (Loghman Pharmaceuticals Company, Tehran, Iran) was injected intramuscularly daily for 3 days. There were no infections, seromas, or hematomas at the recipient sites during the postoperative period. Two months after surgery, all rabbits were euthanized by the lethal dose of thiopental sodium (150 mg /kg, Sigma-Aldrich Company, Germany).

**Fig. 2:**
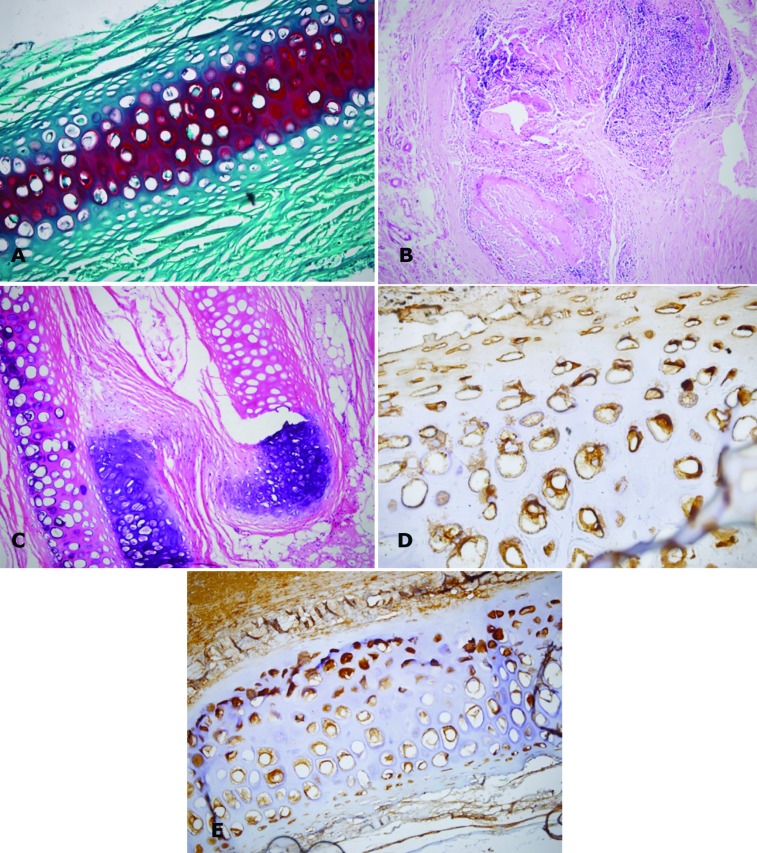
Histology of the different shapes of cartilages with different staining. Matrix demonstrates Safranin-O uptake by the matrix, which is an important evidence of viability of the diced fragments of HAM wrapped in autograft (Safranin-O, ×200) (A). Complete cartilage resorption and replacement by inflamed and neovascularized fibrous tissue in block and crushed heterograft (H&E, ×200, ×400) (B and C). Fibroblast prolifation in diced unwrapped allograft (Verhoeff–Van Gieson, ×200) (D). Positive immunoreactivity for Glial fibrillary acidic protein in crushed HAM Wrapped autogrft cartilage indicating a regeneration capacity (Verhoeff–Van Gieson, ×400) (E)

For histopathological and immunohistochemical analysis, from the neck to sacral region, the entire soft tissue graft of dorsum was elevated as a flap. After completely elevating the flap, the graft sites were excised immediately and sent to the pathologist. All specimens were prepared with histological methods. They were fixed in 10% formaldehyde for 72 hours. All of the specimens were evaluated by histochemical and immunohistochemical staining methods. After preparation of 3-µm slide sections staining with hematoxyline and eosin (H&E; Figure 2B and C), Masson’s trichrome, Verhoeff Van Gieson (Figure 2D and E), and Safranin-O (Figure 2A) were performed (Table 1). 

**Table 1:** Information about different types of staining in specimens

**Stain type**	**Substance stained**	**Color**	**Significance**
Hematoxylin and Eosin	Nuclear chromatin and lacunar glycosaminoglycans	Dark blue	Viable chondrocytes maintain nucleated lacunae
Safranin-o	Proteoglycan content	Red	Viable chondrocytes produce proteoglycan matrix
Masson’s trichrome	Collagen content	Green	Used to compare with normal cartilage
Van Gieson	Elastic fibers	Brown	Normal component of cartilage; for comparison across groups
Glial fibrillary acidic protein	Intermediate filaments	Brown in cytoplasm	Part of a mechanotransduction system by which potentially regenerative cells respond

Also, the specimens were immunostained for glial fibrillary acidic protein (GFAP) by immunoperoxidase method (GFAP Ab, DAKO, RTU, clone 6F2); all compounds were obtained from Sigma-Aldrich Company (USA). Masson’s trichrome was used to identify collagen contents and distribution along the fibrillary matrix of the cartilages. Collagen fibrils were stained bluish in this technique. To assess the content and distribution of amorphous proteoglycan contents of the matrix, Safranin-O was used, and with this technique, proteoglycans were stained in red color. 

Another important component of the soft tissue matrix is elastic fibers that needed to be assessed in this study. These fibers allow tissues to stretch, which cannot typically be seen on routine H&E; thus, we used Verhoeff Van Gieson stain that is specific for elastic fibers. Glial fibrillary acidic protein as a biomarker of the regeneration potential of chondrocytes was used by the immunohistochemical method. Cytoplasmic immunoreactivity of chondrocytes demonstrated the regeneration capacity of chondrocytes. Mature chondrocytes were GFAP-negative and without regeneration. At first, macroscopic findings were detected by pathologists with the aid of the macroscopic grid, such as frank necrosis, discoloration, and change in size, distortion, and atrophy. Histopathologic findings for each group were recorded, and the criteria related to fibroblast proliferation, inflammation, and neovascularization were assessed. The findings for each group were recorded. The selected variables included fibroblast proliferation, neovascularization and inflammation, and classifications were similar to the study of Tarhan et al.^1^

Meanwhile, the presence and severity of inflammation (I1: no or mild inflammation [less than 25 inflammatory cells per lpf], I2: moderate inflammation [more than 25 and less than 50 inflammatory cells per lpf], and I3: severe inflammation [more than 50 inflammatory cells]), the presence and degree of neovascularization (V1: less than 25 microvessels per lpf, V2: 25-50 microvessels per lpf, and V3: more than 50 microvessels per lpf), and finally, the presence and degree of fibrosis (F0: no fibroblastic proliferation and normal collagen in morphologic aspect, F1: mild fibroblastic proliferation with mild irregularity of collagen bundles, and F2: moderate to severe fibroblastic proliferation) were evaluated by one observer, and the pathologist filled the pathological sample sheet. Viability was assessed by the grading system of biopsy and histopathological findings that have been scored from 1 to 5 (Table 2). All findings were compared using the one-way analysis of variance experimental design models using SPSS (Version 22, SPSS Inc., Chicago, IL, USA). Software R3.1.2 was used for the visualization of results. Results were considered to be significant with the P value ≤0.05.

**Table 2:** Viability description and grading system

**Grade**	**Description**
1	Complete resorption, severe bone formation
2	Multifocal resorption >50% or moderate bone formation
3	Focal resorption <50% or minimal bone formation
4	Point resorption <10%
5	Viable tissue, no resorption

## RESULTS

Using HAM could increase the vascularity, proliferation, and viability and decrease in inflammatory. Table 3 shows the amount of every four outcome by three groups of grafts and by using three methods (block, crush, and dice), and using HAM. In the autograft and allograft groups, the dice method had the most inflammatory cells, and the block method had the least inflammatory cells. There were no differences in the heterograft groups (Table 3). Using HAM in the dice method in all grafts had no totally difference in proliferation. The block method had the most proliferation, and using HAM in it increased the amount of proliferation (Table 3).

**Table 3:** The amount of every 4 outcomes by 3 groups of graft and 3 shapes of cartilage (Each square describes a rabbit)

**Viability**																								
**Type**	**Block**								**Crush**								**Dice**							
	**AM-**				**AM+**				**AM-**				**AM+**				**AM-**				**AM+**			
Autograft	4	4	5	5	5	5	4	5	3	4	3	3	4	4	3	5	2	3	2	2	4	3	3	4
Allograft	4	4	5	4	4	5	5	3	2	3	3	2	3	2	4	4	3	2	1	2	3	2	3	2
Heterograft	3	3	3	4	4	5	4	4	2	1	2	2	2	2	2	3	2	1	1	1	2	1	1	2
Proliferation																								
Autograft	1	2	1	1	2	2	2	2	1	2	1	2	2	2	1	2	2	1	1	2	2	1	2	1
Allograft	1	2	1	2	2	2	1	2	2	1	1	1	2	1	1	2	1	1	1	1	1	1	2	2
Heterograft	1	1	2	1	1	1	2	1	1	1	1	1	1	1	2	1	1	1	1	2	2	1	1	2
Vascularity																								
Autograft	2	3	2	2	3	2	2	3	1	2	1	1	3	2	3	3	2	2	1	1	3	2	3	2
Allograft	3	2	2	3	3	3	2	3	3	2	3	3	3	3	3	3	2	3	2	2	3	2	2	3
Heterograft	1	1	2	2	2	1	1	2	1	2	1	1	2	1	2	2	1	2	1	1	2	1	2	1
Inflammatory																								
Autograft	1	1	1	2	1	2	1	1	2	1	3	1	2	1	1	2	3	2	3	3	3	2	2	3
Allograft	1	1	1	2	2	1	1	2	2	2	1	2	1	1	2	3	3	3	2	2	2	3	2	3
Heterograft	1	2	2	1	2	2	1	2	2	2	1	3	2	2	1	2	1	2	1	2	2	1	2	2

Although HAM caused the increase in vascularity, in all the methods, there was no significant difference in all the grafts (*p*=0.066). The allograft group had the most vascularity than other groups. The block method caused significantly the most increase in viability in all graft groups. Between the crush and dice methods, no significant differences were found in all the grafts. In this study 1 rabbit in every group died during the process and were excluded from the study. The viability of the block and crushed cartilages with HAM was significantly better than groups without HAM (*p*<0.001). 

Almost in all specimens, the autograft and allograft specimens were better than heterograft in terms of viability. The prognosis and absorption of autograft specimens in block shape with HAM were significantly better than others with HAM (*p*=0.031) and without HAM (*p*=0.044). The proliferation rate of fibroblasts in the autograft and allograft specimens was more than that of the heterograft specimens with HAM. Block and crushed shapes of cartilages without HAM had more proliferation of fibroblasts. The number of microvessels per unit in the block and crushed cartilages with HAM was more than 50, but in the case of the diced shape with HAM, it was between 25 and 50. Among all the groups and forms that were bare and wrapped shapes, the block-shaped autografts with HAM had the highest grade of viability with no resorption (*p*=0.042). 

## DISCUSSION

HAM is the innermost semitransparent, thin, elastic layer of the fetal tissue with a thick basement membrane. Over the last few decades, it has been gaining popularity among surgeons because of its unique properties and structure. Clinical application and research on these fetus waste tissues are easily accessible with no ethical problems. Epithelial cells’ migration and differentiation, antibacterial activities, and modulate stromal scarring lead to vast applications of HAM in treatment of burns and ocular surface reconstruction.^19^ Moreover, it has been found to be a rich and valuable source of MSCs and therefore has been proposed in cellular therapy and regenerative medicine.^12^^,^^14^^,^^17^^,^^20^

Moreover, HAM is a potential source of pluripotent cells that can influence to produce specifically hyaline-like cartilage and chondrogenesis in defects in animal models.^20^^,^^21^ Tissues related to the fetus, such as the amnion membrane and fluid; also have low immunogenicity and anti-inflammatory properties that produce only minimal immune reaction.^19^^,^^22^ A study has been conducted based on the potential use of HAM as a scaffold for the repair of the cartilage in patients with osteoarthritis. Similar to our study, the effective and functional application of HAM has been presented in this study. It has been found that the nutrients present in HAM make it feasible to use it as a supportive substance to improve the proliferation of chondrocytes to cellular grafting therapy in osteoarthritis patients’ cartilage. HAM has antimicrobial, antifibrosis, antiangiogenic, and antitumorigenic properties. It also reduces inflammation and inflammatory cells and scars, promotes wound healing and epithelialization, and is used as an anatomical and vapor barrier.^18^

Also, Jorge *et al.* demonstrated whether acellular HAM engraftment could improve reconstruction of partial tracheal defects in both the macroscopic and microscopic levels. These results indicate that acellular HAM engraftment could facilitate neovascularization and regeneration of immature cartilage in a model of tracheal injury. Its use may decrease the risk of postoperative complications including stenosis of trachea.^23^ We have determined that the proliferation of chondrocytes, vascularity, and viability in block shapes with HAM in all types of grafts was more than those in others. 

Recent studies have suggested that MSCs isolated from the fetus, related to tissues, have the ability for tissue regeneration, immune modulator in transplantation tolerance and autoimmunity. Some authors have shown that the umbilical cord MSC do not require tissue matching; therefore, any donor can give cells to any person without rejection or need of immunosuppressive drugs.^24^ Some studies presented dehydrated HAM provides an alternative to local tissue transfer and skin grafting for traumatic injuries involving the nose.^25^^,^^26^

The effects of wrapping with surgicel or fascia are the main subject of discussion in many articles. The procedure of wrapping in the cartilage graft for plastic surgery was first stated by Erol in 2000, by utilizing surgicel with the diced cartilage to improve contour deformities and graft prognosis, as reputed “Turkish Delight”.^27^ In 2004, Daniel *et al.* replaced surgicel by fascia. Both materials may lead to interference with graft taking. Surgicel stimulates the host immune system and causes inflammation and cartilage resorption within 6 months. In contrast, in fascia, the prognosis of wrapped grafts is much longer than that in surgical.^28^ Some authors have suggested that fascia disrupts the plasmatic diffusion to cartilage.^29^

Firat *et al.* showed that allograft without perichondrium is similar to autograft with host tolerance, and low antigenicity in an animal study also mentioned that the prognosis and structure of bare diced and block grafts were better than fascia and surgicel wrapped forms.^29^ Also, our study has shown that inflammation and fibrosis in dice shapes with and without HAM were the most, and we had the lowest inflammation in block and crushed shapes of cartilages with HAM in autografts and allografts. This probably could be related to HAM’s anti-inflammatory and antifibrosis effects. The fate of surgicel is early degradation and systematic clearance by phagocytosis.^30^

Covering materials as temporalis fascia^5^ and dermal grafts^6^ have been studied by some authors for the second time, and the correction of deformities, but late deformities, were due to volume reduction.^31^ Tarhan *et al.*^1^ concluded that, among various graft materials in the rabbit’s model, including autologous cartilage, dermal tissue fat, and alloderm, the best graft material is cartilage, followed by fascia with a minimal shrinkage capacity and tissue reaction. In another study, the effects of adipose-derived stem cells on diced cartilage grafts showed improvement in the graft tissue viability.^32^

Although there is similarity with our study, fat harvesting in humans is an invasive procedure. Our study used a technique similar to the one used in Yilmaz *et al.*,^33^ but instead of surgicel, we used HAM as biological coverage. The result of this study indicated that wrapped diced grafts have high cartilage proliferation with positive effects on cartilage viability and regeneration. Such findings have also been presented by other authors. Overall, the use of oxidized regenerated cellulose to wrap diced cartilage grafts also tends to reduce clinical predictability. Some authors advocated that deep temporal fascia is the preferred envelope than surgicel, with improved diced graft prognosis being associated with use of temporal fascia.^34^

In this study, macroscopic atrophy and microscopic changes were determined to be identical among autografts and allografts, with the exception of diced allografts that had partial resorption in bare grafts. This issue was similar to the research by Firat *et al.*^29^ which they believed to have antigenicity power of perichondrium. If it removed from the surface of the graft, it lead to the improvement of allograft outcome. Our study like Wei *et al.*’s study showed that human amniotic mesenchymal cells had the potential to differentiate into chondrocytes in vitro and in vivo, suggesting that they have therapeutic potential for the treatment of damaged or diseased cartilage.^35^

The limitations of this study were hard work conditions for keeping the rabbits. Because of this, 3 rabbits died during the process and our sample size was approximately low. Strength points of it were study on effect of the human amniotic membrane on the cartilage itself, different types of cartilage, and different shapes in each of the cartilage types. On the other hand, the clinical use of various types and shapes of cartilage in the aesthetic and restorative nasal surgeries is very high, as plastic surgeons often deal with this issue daily. Cellular therapy has emerged as a new solution with considerable effects in many plastic surgery procedures. 

The results of this study presented a new aspect of HAM application as the alternative source of stem cells, which can facilitate an improvement in graft prognosis. We believe that HAM can improve graft quality with increased blood circulation. However, infection, distortion, warping, and resorption of cartilage grafts are probably decreased by HAM. Our findings suggest that HAM as biocompatible and biologic coverage may lead to the reduction the absorption of grafts, as well as minimal tissue fibrosis, inflammation, disfiguring, and promotion of neovascularization in some cases. In future, these fetus materials can be a suitable alternative for surgicel, fascia, and alloderm. This study shows a few promising signs, indicating that HAM may have effects on the viability of cartilage grafts. It is necessary to design other studies with the application of multilayer wrapping of HAM. The behavior of grafts and HAM in animals may differ from humans, but the primitive result from this research may encourage other researchers to design other studies for more definitive results.
